# The benefits of banks’ IT investments in times of trouble: evidence from loan loss accruals during the COVID-19 pandemic

**DOI:** 10.1007/s11573-022-01100-0

**Published:** 2022-06-16

**Authors:** Moritz Sefried, Jan Riepe

**Affiliations:** Department of Banking, University of Tübingen, Nauklerstr. 47, 72074 Tübingen, Germany

**Keywords:** Bank accounting, Loan loss provisions, IT investments, COVID-19 pandemic, G21, G32, M41

## Abstract

Motivated by diverging results from the literature, we investigate whether investments in information technology (IT) help banks to assess their loan portfolio. More specifically, we focus on the consequences of accumulated expenses for data processing on banks’ ability to estimate their loan loss accruals. We further test for differences when the banks’ borrowers get hit by the economic trouble from the COVID-19 pandemic. Using a sample of US commercial banks before and during the COVID-19 pandemic, we find more precise estimates of loan loss accruals during these troublesome times in banks that accumulated higher data processing expenses. Surprisingly, we do not find significant differences in the precision of loan loss accruals by banks’ IT investments during normal times. Our findings contribute to consolidate previously diverging results by showing that IT investments help banks following a structural break, such as the COVID-19 pandemic.

## Introduction

Information technology (IT) has become increasingly important to banks. Banks have spent heavily on IT, for example, by developing a clear digital strategy, redesigning core processes, and making significant investments in IT infrastructure. Appropriate IT systems do not only help banks save time and effort in the faster extraction of more fine-grained information, but they are also a vital resource in assessing risks within the banks’ core business activities (Grasshoff et al. 2019; Hauswald and Marquez 2003).

The COVID-19 pandemic that spread throughout the world in 2020 has made efficient information processing even more pressing because expectations for and outlooks on the clients’ economic wellbeing have become highly volatile, and banks have had to question previously identified trends each and every day. This holds true for firms in general and banking firms specifically. The OECD (2020) highlights the importance of adapting to the new circumstances coming with the pandemic. If firms do not sufficiently invest in IT and do not implement IT appropriately, they would risk falling behind in terms of productivity (OECD 2020). In a similar vein, Andrea Enria, Chair of the Supervisory Board of the ECB, argues that digitalization does not only help banks enhance their revenues but also improve banks’ cost-efficiency (Enria [ECB] 2021). According to the IDC survey, bank managers were also highly aware of the importance of IT systems during the COVID-19 pandemic and have prioritized process automation in the face of spiking workloads as a result of the COVID-19 crisis (Reuters 2020). Furthermore, the internal business processes within banks face new challenges, including lockdowns and remote work from home, which is easier to cope with for banks with higher IT expertise.^1^ The build-up of IT expertise thereby did not happen overnight but required long-term investments. Jerry Silva, global banking research director at IDC, refert to a digital divide in the banking industry during the Covid-19 pandemic: "Sometimes I call it the predatory gap, because those banks are going to be able to steal market share from those that were not prepared prior to 2020.” (Reuters 2020).

However, our understanding of the bank-level consequences of better IT is limited. The research frequently fails to isolate the direct implications of higher IT investments for performance, and the empirical evidence on these implications strongly diverges (Beccalli 2007; DeYoung et al. 2007; Koetter and Noth 2013; Buchak et al. 2018; Pierri and Timmer 2020). Plausible reasons for the inconclusive evidence are a lack of proper data as well as a well-suited empirical identification strategy (Beccalli 2007; DeYoung et al. 2007; Buchak et al. 2018).

In this study, we investigate the link between IT investments and the internal business processes in banks during the COVID-19 pandemic. Our study investigates how IT capabilities are helpful for banks during the COVID-19 pandemic in coping with and managing loan risk after a loan was already granted to the borrowers. Therefore, our investigation uses the absolute abnormal loan loss provisions (*|ALLP|*)^2^ as main proxy (Dal Maso et al. 2018). We investigate banks because they have heavily invested in information technologies and have become technology-intensive (Berger 2003; Beccalli 2007). Consequently, the bank-level consequences of IT investments are highly meaningful and relevant. We explore the COVID-19 pandemic because it provides a unique empirical identification. The COVID-19 pandemic represents a plausibly exogenous shock to banks’ information quality when screening and monitoring borrowers. Because different industries got hit by the pandemic in remarkably different ways and indirect effects from the highly volatile stock market affected borrowers’ collateral and liquidity needs, the economic situations of borrowers during the pandemic changed more frequently and in less predictable ways. Duan et al. (2021) provide empirical support for the effect of the COVID-19 pandemic on loan risk. Moreover, Pierri and Timmer (2021) study consumer spendings during the COVID-19 pandemic and find that IT can play an important role as a mitigating (short-term) factor when a pandemic hits the economy. By using a database with information from telephone research interviews, Kwan et al. (2020) study the effect of IT capabilities on banks’ ability to serve customers. They claim that banks with higher IT capabilities are, on the one hand, better positioned for the future, where probably fewer bank branches and more digital banking are present, and also for extreme shocks like the COVID-19 pandemic itself.

Overall, we expect IT investments to positively correlate with banks’ quality of loan risk assessments. Especially in a field where data have such pivotal importance, banks’ reliance on well-implemented processes is key to their decision-making. Since the amount of data continues to grow, having IT systems that can handle this data is critical. In times of relatively stable economic conditions, banks can use their experience to estimate future loan losses and, thus, build up accruals based on that experience. If this is the case, IT investments could still be helpful in decision-making but not crucial since banks’ managers can orient themselves on the values from previous periods. However, and following the literature on the business level consequences of better IT capabilities in banks, we expect that banks with better IT capabilities more precisely estimate their loan loss provisions in general. Moreover, we use the COVID-19 pandemic as a shock to banks’ information environment and argue that banks with better IT capabilities could better cope with that shock. Thereby, our identification relates to the approach by Pierri and Timmer (2020), who also use a shock to identify the bank-level consequences of banks’ IT capabilities. In contrast to Pierri and Timmer (2020), we do not use a shock to the financial system and the regulatory capital from the 2008 financial crisis, but we rely on the COVID-19 pandemic that primarily influences the banks’ clients but not directly the financial sector. Nevertheless, and in line with the results from Pierri and Timmer (2020) and due to banks’ capability to process data quicker and in more detail (Hauswald and Marquez 2003; Grasshoff et al. 2019), we expect banks with higher levels of IT investments do better assess their (loan) risk in highly uncertain times compared to banks with lower IT investments. Since the pandemic forces banks to adapt quickly to a new business environment, we expect that data processing is even more advantageous in such unstable times. Additionally, when this pandemic occurred, banks with lower IT investments could not use their experience on loan losses during stable times before but needed to make new assumptions. During those times, IT investments are, consequently, most valuable.

We find that banks with higher IT capabilities can better assess their loan risk in times of the pandemic or, more generally, in times of high uncertainty after a structural break. In our setting, this is true for the first two quarters of the year 2020. Afterwards, the influence reduces and it comes to an alignment of banks with high and low IT capabilities. Surprisingly, however, IT capabilities do not seem to play a significant role in stable times, which we hark to the Bayesian learning theory.

Our study relates and contributes to the literature in different ways. Our study closely relates to Beccalli (2007). She investigates whether banks’ IT investments improve their performance using a sample of 737 commercial banks from Europe in the pre-dotcom era. She only finds a very weak and partially negative link between banks’ IT spending and profitability. Based on her findings, she articulates a “profitability paradox”. In contrast to Beccalli (2007), who relies on expert estimates of IT investments and some voluntary disclosures on IT investment, we use mandatory data from the FDIC on all commercial banks in the United States (US). We further relate to DeYoung et al. (2007). They study the consequences of banks’ internet adoption on different balance sheet and income statement items based on a sample of US commercial banks around the turn of the millennium. They find a positive link between early internet adoption and current profitability. Koetter and Noth (2013) use banks’ productivity to measure performance rather than the net income. Koetter and Noth (2013) measure IT expenditures as the sum of costs for software, hardware, third-party services, shared service centers, and information transmissions. They use a dataset that comprises over 400 German savings banks between 1996 and 2006. Estimating this relationship with five different alternative output definitions, they find a significant and positive contribution of IT investments to banks’ output and conclude that IT can help improve the screening and monitoring of banks’ borrowers. Since data on IT investments is hard to gather and frequently neither publicly available nor structured and detailed, Kriebel and Debener (2019) try to measure banks’ IT investments by examining their annual reports with a textual analysis. They find a positive link between more IT-related words in annual reports and items in the income statement. In other related work, Buchak et al. (2018) investigate the consequences of IT on FinTechs’ growth, Fuster et al. (2019) on FinTechs loan processing abilities, and Di Maggio and Yao (2020) on FinTech’s loan screening ability. Fu and Mishra (2022) study finance-related mobile app market in times of the COVID-19 pandemic.

The work of Pierri and Timmer (2020) was one of the first studies on the risk-consequences of better IT capabilities and, consequently, very closely relates to our study. Pierri and Timmer (2020) investigate banks’ loan quality around the 2008 financial crisis for a sample of US commercial banks. Pierri and Timmer (2020) find evidence that banks with more IT capabilities (as measured by the share of personal computers in that bank) were able to select more solvent borrowers. However, our study complements the work by Pierri and Timmer (2020) by focusing more on monitoring loans rather than screening customers, which means that loans have already been granted to the customers in our setting.

We contribute to the literature by providing new evidence on the performance level consequences of IT capabilities on banks’ business using standardized data by the FDIC. We contribute to the literature by providing new empirical evidence on the relation between IT investments and the quality of risk assessments in banks with an advantageous setting and dataset.

Thereby, we also relate to the work by Berg et al. (2020) that investigates one underlying channel that helps to explain a link between banks’ IT capabilities and their loan defaults. They analyze whether banks can use their customers’ digital footprint to better forecast their likelihood of default on loan obligations. In addition to a credit bureau score, Berg et al. (2020) find that the digital footprint can indeed support the company in its lending decisions.

## Methodology

### Measurement of information technology investments

The empirical research on the consequences of banks’ IT investments suffers from a lack of IT expense information in the popular databases. Furthermore, strong endogeneity concerns regarding the direction of the relationship (Koetter and Noth 2013) call for comprehensive data and a suitable empirical setting. Compared to other countries, a huge advantage of the US banking sector is that IT expense data is available and disentangled on bank-level and not on group-level. Additionally, the level of service and the customer approaches of smaller commercial banks in the US are very similar to those in other parts of the world. Therefore, we are convinced that our results can be transferred to group-level and consequently other countries.

Banks have improved their capabilities in terms of information technologies and can use them to their economic advantage. Bharadwaj (2000) calls this capability a firm’s IT capability and defines it as the “ability to mobilize and deploy IT-based resources in combination or copresent with other resources and capabilities”. Bhatt and Grover (2005) split capability into three different types: value, competitive, and dynamic. Value capability refers to firms’ IT investments; competitive capability refers to firms’ IT business experience and the relationship between IT and business managers; and dynamic capability refers to the firm’s knowledge about and adaptation to technological changes and new opportunities. In this study, we mainly refer to banks’ value capability for IT in the sense of Bhatt and Grover (2005) as our empirical proxy measures the accumulated investments in banks’ data processing. Hereafter, we will use the term IT capability (Bharadwaj 2000) when discussing the underlying concept of how IT influences banks’ business activities and the term IT investments when referring to our empirical measure for data processing-related IT capability.

Nevertheless, the measurement of banks’ IT capabilities still is a crucial challenge for empirical research. This study relies on banks’ investment in IT and specifically the data processing expenses from their quarterly call reports to measure IT capabilities. The link between bank IT capability and banks’ IT investments has been frequently argued (Bhatt and Grover 2005) and used in previous empirical literature (Beccalli 2007; Koetter and Noth 2013; Xin and Choudhary 2019). The argument follows the idea that banks can acquire IT capabilities by investing in commercially available IT, which means IT adoption is not exclusive (Xin and Choudhary 2019). In our main specification, we directly refer to those IT capabilities that are closely related to data processing, as this is closer to the actual acquisition of IT capabilities. Data processing expenses is a mandatory separate line item in banks’ quarterly income statements in the other non-interest income section.

The use of quarterly information thereby allows us to capture changes in banks’ IT capabilities over time. Using such a time-variant measure for IT capabilities is one innovation in this study. It enables us to fully use our panel data that is impossible when using other frequently used proxies like a snapshot of a ranking or a cross-sectional survey (Bharadwaj 2000). In this way, we can measure whether changes in banks’ IT investments have business consequences. We can thereby rule out biases from the time-invariant factors that simultaneously influence banks’ IT capabilities, such as geographic or institutional factors.

Nevertheless, we openly acknowledge that past IT investments do not perfectly predict current IT capabilities because IT projects might fail (Xin and Choudhary 2019). Second, specific IT hardware and knowledge might take some time to be adequately implemented (Campbell 2012). But at the same time, IT hardware loses its value over time because of technological change. Failing IT projects will thereby create noise to our measure and bias our empirical results against finding anything. Therefore, our empirical evidence for our measure is a conservative estimate of the actual underlying relationship. Furthermore, we explicitly use IT investments related to data processing to mitigate the effects of large investments in banks’ IT infrastructure, whose failure directly results in the recognition of the expenses on their balance sheets. Technological change and the implementation time call for the use of lagged information on banks’ IT investments but require accumulating those investments for only a few quarters. In this study, we decided to use the average IT investments from the first quarter of the year 2015 to the last quarter of 2019, just before the COVID-19 Pandemic started, as our main explanatory variable. Nevertheless, our empirical inferences remain qualitatively unchanged if we use the average IT investments from 2017, or a rolling IT measure as seen in the robustness checks in Chapter 5.

### Testing the relationship between IT investments and quality of banks’ loan risk assessments

LLPs are banks’ most important, loan-related accrual (Liu and Ryan 2006; Kanagaretnam et al. 2010b; Beatty and Liao 2014). On average, IT investments are banks’ third-largest non-interest expense and are just as large as their marketing expenses, legal fees, accounting, and consulting expenses taken together. Banks’ loan loss provisions (LLP) are economically important because they are their largest accrual and are tied to a broad range of other outcomes (Beatty and Liao 2014). Studies have frequently used them to measure banks’ transparency or earnings quality (Kanagaretnam et al. 2010a; Beatty and Liao 2014; Jin et al. 2018) since bank managers can and potentially want to engage in steering LLP. This steering might reduce the overall quality of earnings (Jin et al. 2021). We control for this discretionary behavior by adding control variables to our LLP specification. Afterward, we can determine the absolute misestimation of banks’ LLP, which we refer to as the quality of risk assessments. Thus, the more accurately banks assess their LLP, the higher the quality of risk assessment we perceive for these banks.

We closely follow the two-step approach from Jin et al. (2021) and Dal Maso et al. (2018) and concentrate on the magnitude of abnormal LLP to represent the quality of banks’ loan risk assessments. First, we estimate the LLP for each quarter using an LLP model, which closely follows Beatty and Liao (2014). We extend their model by adding *EBLLP*, *RegCap*, and *LLA* to account for manager discretion. Overall, our first stage regression is as follows:

1 $${LLP}_{i, t}= {\beta }_{0}+ {\beta }_{1}{dNPL}_{i,t}+ {\beta }_{2}{dNPL}_{i,t-1}+ {\beta }_{3}{RegCap}_{i,t-1}+ {\beta }_{4}{CO}_{i,t}+ {\beta }_{5}{CO}_{i,t-1}+ {\beta }_{6}{EBLLP}_{i,t}+ {\beta }_{7}{dLoans}_{i,t}+ {\beta }_{8}{LLA}_{i,t-1}+ {\beta }_{9}{Size}_{i,t-1}+ {\alpha }_{j},$$

where ${LLP}_{i, t}$ stands for the loan loss provisions scaled by total loans of the bank, ${dNPL}_{i, t}$ is the change in nonperforming loans from the previous to the current year at the bank level that is divided by total loans, and ${RegCap}_{i,t-1}$ is the previous year’s share of regulatory capital that is scaled by risk-weighted assets. ${CO}_{i,t}$ represents the ratio of charge-offs in the current year to loan loss allowances, and ${EBLLP}_{i,t}$ represents the earnings before loan loss provisions that are scaled by total loans. ${dLoans}_{i,t}$ is the change in loans from the previous to the current year that is scaled by total assets, ${LLA}_{i,t-1}$ stands for the amount of loan loss allowances in the previous year that is divided by total loans, and ${Size}_{i,t-1}$ represents the natural logarithm of total assets from the beginning of the period. The values of the profit and loss statement variables reflect the actual amount added in the respective quarters. Further, ${\alpha }_{j}$ represents the fixed effect on the state-level. Technically, we conduct a regression for each quarter itself. In this way, we have a regression model that is equivalent to a state-by-time fixed effects model since the respective coefficients can vary for each quarter and, therefore, capture regional-specific influences per period.

In the next step, we calculate *|ALLP|*, the absolute residuals of the first-stage regressions. We use the absolute value because we are not interested in whether banks under- or overestimate their LLP but whether they have any deviations. The *|ALLP|* is our final measure of the quality of banks’ assessments of loan risk. Since we are interested in the relation between banks’ loan risk assessments and IT investments, our main explanatory variables are IT investments from before the pandemic and the interaction term for IT investments during the COVID-19 pandemic. Therefore, after calculating *|ALLP|*, we regress our *IT investments* variable together with call report items on *|ALLP|* to test their relationship. As already mentioned, we include an interaction term for *IT investments* and the year of the pandemic to gather the correlation of *IT investments* during a structural break. Additionally and to account for serial correlation in our model, we cluster the standard errors on bank-level. Consequently, our regression model looks as follows:

2 $$\left|{ALLP}_{i, t}\right|= {\beta }_{0}+ {\beta }_{1}{ IT investments}_{i,t}* {COVID Crisis}_{t}+ {\beta }_{2} {IT investments}_{i,t}+ {\beta }_{3}{COVID Crisis}_{t}+ {\beta }_{4}{EBLLP}_{i,t}+ {\beta }_{5}{RegCap}_{i,t}+ {\beta }_{6}{Asset Growth}_{i,t}+ {\beta }_{7}{Loans to Assets}_{i,t-1}+ {\beta }_{8}{Deposits to Assets}_{i,t}+ {\beta }_{9}{Deposits to Assets}_{i,t-1}+ {\beta }_{10}{dNPL}_{i,t}+ {\beta }_{11}{dNPL}_{i,t-1}+ {\beta }_{12}{Real Estate Loans}_{i,t}+{\beta }_{13}{Commercial Loans}_{i,t} + {\beta }_{14}{Retail Loans}_{i,t}+ {\alpha }_{j}+ {\tau }_{t},$$

where ${IT investments}_{i,t}$ stands for our eight quarter IT measure; the binary variable ${COVID Crisis}_{t}$ represents the year 2020 when the outbreak of COVID-19 occurred. It equals one for each quarter in the year 2020 and zero otherwise.

The literature uses a large set of control variables in a very heterogeneous way. Some variables appear in many studies, while others occur in one or two empirical models (see Beatty and Liao (2014) for a discussion of the differences in the early LLP models). We closely follow Dal Maso et al. (2018) in our selection of control variables. We differentiate by refraining from including constant state variables, but our state-fixed effects account for this exclusion. In a nutshell, we control for bank characteristics with different loan-related variables because they are closely related to the level of LLP and thus allow us to more precisely capture the influence of our explanatory variable *IT investments*. Namely, we include banks’ *EBLLP* because, for example, Kilic et al. (2021) argue that income smoothing is especially important in bank accounting. We also add the ratio of *Regulatory Capital*, lagged *Loans to Assets* ratio, *Deposits to Asset* ratio of the current and prior year, and changes in *NPL* of the past two years as well as *Asset Growth*. We also add the *Real Estate*, *Commercial*, and *Retail Loans* of the respective bank since the manager’s discretion over LLP differs across loan types (Liu and Ryan 1995; Bhat et al. 2014). Further,${\alpha }_{j}$ is the fixed effect on the state level, and ${\tau }_{t}$ is the fixed effect per quarter. The variables mentioned above allow us to control for banks’ specific business focus and reduce the chance of an omitting variable bias in our estimation of the IT investments correlation.

The coefficients of interest are ${\beta }_{1}$ and ${\beta }_{2}$ in Eq. (2). We expect IT investments to reduce *|ALLP|*. Thus, we expect a negative coefficient for ${\beta }_{2}$. Thereby, it captures the overall link between IT investments and *|ALLP|*. ${\beta }_{1}$ identifyies the differences in the link between IT investments and *|ALLP|* during normal and crisis periods. We expect that past IT investments are even more beneficial in reducing *|ALLP|* during crisis times that should lead to a negative ${\beta }_{1}$. As articulated by Dal Maso et al. (2018), the error terms from Eq. (1) are used in Eq. (2) and thus can influence the coefficient estimates (especially by extreme values). To address this concern, we use a robust regression estimation.

## Data

We use quarterly call reports from the Federal Financial Institutions Examination Council (FFIEC) for all US commercial banks in the period from 2015 to 2020. We drop bank observations with missing or negative values on core balance sheet items, such as equity, total assets, and IT investments.^3^ Additionally, we limit our sample to commercial banks because LLP only provide meaningful information on the business-level consequences of IT investments for these firms. We especially eliminate financial service firms and investment banks with a loan to asset ratio or deposits to total assets ratio below 50%. We drop small banks with total assets below 100,000 USD because small banks operate in a different regulatory environment. Furthermore, we delete observations characterized by extensive merger activities and financial distress to limit any confounding effects from corporate restructuring on the bank-level outcomes. After cleaning up our sample, we have 8,522 observations from 665 banks with data on all variables left for our regressions. The remaining banks in our sample are typical commercial banks. To further account for outliers, we winsorize all variables at the 1% level.

## Empirical results

### Univariate

The descriptive statistics of all variables are provided in Table 1. Banks in our sample have, on average, 84.7% of deposits and 74.6% of loans on their balance sheets. Our primary variable of interest is *IT investments* which is a line-item of other non-interest expenses. We scale IT investments by banks’ total other non-interest expenses to alleviate competing influences from banks’ loan portfolio, that is, LLP and charge-offs. There are considerable differences in our measure of IT investments for each bank. While banks from the lower quantile have on average a ratio below 13%, banks belonging to the highest quantile have on average a ratio of more than 25%.

**Table 1:** Descriptive statistics and correlation matrix

	Mean	Std. Dev	p25	p50	p75	(1)	(2)	(3)	(4)	(5)	(6)	(7)	(8)	(9)	(10)	(11)	(12)	(13)	(14)	(15)	(16)	(17)
1^st^ stage																						
(1) *LLP*	0.001	0.001	0.000	0.000	0.001																	
(2) *dNPL*	0.000	0.005	− 0.001	− 0.000	0.001	0.08*																
(3) *RegCap*	0.138	0.035	0.116	0.129	0.147	− 0.02*	− 0.05*															
(4) *CO*	0.039	0.073	0.003	0.015	0.042	0.51*	0.01	− 0.05*														
(5) *EBLLP*	0.016	0.013	0.012	0.017	0.023	− 0.66*	− 0.06*	0.07*	− 0.36*													
(6) *dLoans*	0.015	0.028	-0.001	0.011	0.024	0.05*	0.02	− 0.13*	− 0.04*	− 0.00												
(7) *LLA*	0.012	0.005	0.009	0.011	0.014	0.45*	− 0.02	0.12*	0.10*	− 0.17*	− 0.14*											
(8) *Size*	13.954	1.350	12.923	13.892	14.773	0.15*	0.06*	− 0.24*	0.12*	− 0.01	0.08*	− 0.13*										
2^nd^ stage																						
(9) *ALLP*	− 0.035	6.158	− 3.434	− 0.156	2.968	0.45*	− 0.01	0.00	− 0.03*	0.03*	0.01	0.05*	0.01									
(10) *|ALLP|*	4.460	4.671	1.428	3.208	5.889	0.42*	0.06*	0.08*	0.28*	− 0.26*	0.03*	0.22*	0.05*	0.14*								
(11) *IT Investments*	0.189	0.086	0.127	0.188	0.245	-0.04*	0.03*	0.04*	− 0.05*	0.01	0.00	− 0.03*	− 0.18*	0.01	− 0.05*							
(12) *COVID Crisis*	0.227	0.419	0.000	0.000	0.000	0.21*	0.09*	0.01	− 0.04*	− 0.16*	0.05*	0.10*	0.11*	0.00	0.23*	− 0.04*						
(13) *Asset Growth*	0.024	0.046	− 0.000	0.016	0.036	0.08*	0.04*	− 0.10*	− 0.01	− 0.02	0.67*	− 0.08*	0.08*	0.02	0.05*	0.01	0.22*					
(14) *Loans to Assets*	0.746	0.088	0.685	0.749	0.810	0.06*	0.06*	− 0.26*	0.02	− 0.14*	0.40*	− 0.16*	0.08*	− 0.02	− 0.02	0.03*	− 0.04*	0.31*				
(15) *Deposits to Assets*	0.847	0.067	0.808	0.852	0.889	0.01	0.02	− 0.18*	− 0.04*	0.05*	0.29*	0.08*	− 0.22*	0.03*	− 0.02	0.06*	0.15*	0.52*	0.03*			
(16) *Real Estate Loans*	0.521	0.166	0.418	0.533	0.634	− 0.23*	− 0.04*	0.06*	− 0.17*	− 0.02	0.16*	− 0.36*	0.08*	− 0.06*	− 0.10*	− 0.07*	− 0.07*	0.10*	0.39*	− 0.14*		
(17) *Commercial Loans*	0.115	0.080	0.058	0.097	0.151	0.08*	0.07*	− 0.36*	0.06*	− 0.02*	0.19*	− 0.04*	0.16*	0.03*	0.04*	0.02*	0.21*	0.20*	0.19*	0.20*	− 0.31*	
(18) *Retail Loans*	0.033	0.054	0.004	0.013	0.033	0.34*	0.01	− 0.04*	0.29*	− 0.16*	0.00	0.20*	0.09*	0.07*	0.10*	0.03*	− 0.03*	0.00	0.03*	0.03*	− 0.38*	0.01

For representational reasons, *ALLP* and *|ALLP|* in Tables 1 to 5 are multiplied by 10^4

*p < 0.05

Table 1 provides the summary statistics on the left-hand side of the table−columns (1) – (17) state the Pearson correlation coefficients between the variables. Continuous variables are winsorized at the 1% level

Table 1 also displays the pairwise correlations of IT investments and other bank characteristics. The correlations do not indicate any issues related to multi-collinearity and are all in line with our expectations. We find a negative correlation between *IT investments* and *Size* that indicates that IT investments are easily scalable. *EBLLP* shows no statistically significant correlation with *IT investments,* while *Regulatory Capital* and *IT investments* are positively correlated. The correlations are not in line with IT investments depending on banks’ free cash flow but indicate a more planned and conscious decision by banks on IT investment.

To strengthen our findings from the correlation analysis, we further look at variance inflation factors (VIF) to assure that our variables are not affected by multicollinearity. The highest VIF value in our regression model is 3.87 for the *Deposits to Asset* variable of the current period. Unfortunately, there is in general no critical threshold for the VIF that indicates multicollinearity. As a rule of thumb, a VIF between 5 and 10 or greater than 10 is likely a sign for multicollinearity. Since this is not the case for our regression, the VIF analysis is in line with the results from the correlation analysis.

Table 2 complements the correlation analysis by providing empirical evidence from a regression of IT investments on *|ALLP|* for different time periods. We observe a significant and negative correlation between the IT measure and *|ALLP|* only during the COVID-19 pandemic. For the years before the pandemic, the correlation is negative but far smaller and not statistically significant. The results are not in line with our prediction for stable times but provide initial support for the value of IT in times of high uncertainty.

**Table 2:** Regression of absolute abnormal loan loss provisions on IT investments

Dependent variable	(1)	(2)	(3)
|ALLP|	Pre COVID-19 pandemic	COVID-19 pandemic	Full sample
IT investments x COVID Crisis			− 5.30838**
			(2.11318)
COVID Crisis			3.31228***
			(0.49868)
IT investments	− 1.37245	− 5.87718***	− 1.50134
	(1.11008)	(1.93654)	(1.12459)
State & Time FE	Yes	Yes	Yes
Observations	6591	1931	8522
R-squared	0.13908	0.15173	0.15383

***p < 0.01, **p < 0.05, *p < 0.1

Robust standard errors are in parentheses

Table 2 contains three OLS regression specifications with standard errors that are robust and clustered on bank level. The dependent variable is *|ALLP|*. Column (1) only comprises the Pre COVID-19 crisis timeframe, whereas column (2) only comprises the COVID-19 crisis year, namely, the year 2020. However, column (3) includes the full sample. We regress IT investments solely on *|ALLP|* to test their relationship for the first two specifications. We incorporate an interaction term in column (3) since the Pre COVID-19 crisis and the crisis period itself are included. Additionally, we conduct the regressions with state- and time-fixed effects. Continuous variables are winsorized at the 1% level

### Multivariate

Table 3 shows the results of the OLS estimations of *|ALLP|* on *IT Investments* with control variables. In column (1), we only consider the years before the outbreak of the COVID-19 pandemic. Column (2) is limited to the observations of the pandemic year only, and column (3), our main specification, has all observations since the year 2016 and an interaction term for the COVID-19 pandemic and *IT investments*.

**Table 3:** Regression of absolute abnormal loan loss provisions on IT investments and control variables

Dependent variable	(1)	(2)	(3)
|ALLP|	Pre COVID-19 pandemic	COVID-19 pandemic	Full sample
IT investments x COVID Crisis			− 4.97753**
			(2.08462)
COVID Crisis			3.19506***
			(0.50835)
IT investments	− 1.78099	− 5.19143***	− 1.76565
	(1.12302)	(1.87027)	(1.14386)
*Controls:*			
dNPL	12.31328	78.09683**	26.93091*
	(13.26871)	(33.83121)	(14.05298)
Lagged dNPL	9.47364	32.3366	10.79672
	(13.14489)	(35.19397)	(12.94717)
RegCap	9.76714***	9.31151*	9.01669***
	(2.51508)	(5.64969)	(2.72012)
EBLLP	− 59.90706***	− 113.44291***	− 74.32631***
	(13.94126)	(22.83986)	(15.21513)
Asset Growth	5.98414***	7.42889*	5.2041***
	(2.04721)	(4.39928)	(2.01126)
Lagged Loans to Assets	− 0.68257	− 1.21702	− 1.49504
	(1.20706)	(2.86646)	(1.36502)
Deposits to Assets	− 2.35615	− 12.13982**	− 4.78267**
	(1.8491)	(5.38401)	(2.16516)
Lagged Deposits to Assets	-0.67845	2.79325	0.46479
	(1.43999)	(4.18671)	(1.73982)
Real Estate Loans	− 1.05162	− 1.69304	− 0.80649
	(0.94128)	(1.85896)	(1.00803)
Commercial Loans	− 0.50356	− 0.78833	0.41306
	(1.46567)	(2.55385)	(1.58325)
Retail Loans	0.90592	− 1.80632	− 0.56595
	(2.81759)	(4.61755)	(2.97823)
State & Time FE	Yes	Yes	Yes
Observations	6591	1931	8522
R-squared	0.18091	0.23082	0.19752

***p < 0.01, **p < 0.05, *p < 0.1

Robust standard errors are in parentheses

The table contains three OLS regression specifications with standard errors that are robust and clustered on bank level. The dependent variable is *|ALLP|*. Similar to Table 2, column (1) only comprises the Pre COVID-19 crisis timeframe, whereas column (2) only comprises the COVID-19 crisis year, namely, the year 2020. However, column (3) includes the full sample. Here, we regress IT investments and control variables on *|ALLP|* to test whether our findings from Table 2 still hold. We incorporate an interaction term in column (3) since the Pre COVID-19 crisis and the crisis period itself are included. Again, we conduct the regressions with state- and time-fixed effects.

Column (1) shows *IT investments* have a negative but statistically non-significant coefficient in normal times. The coefficient changes in column (2) and supports our hypothesis that IT investments are more helpful in times of a crisis. Since we excluded the years before the COVID-19 pandemic in column (2), the pandemic simultaneously affects the coefficients for *IT investments* and the control variables. To isolate the incremental benefits of IT investments conditional on the pandemic period, we use the interaction term between *IT investments* and the *COVID Pandemic* dummy. In column (3) of Table 3, we observe a negative and statistically highly significant coefficient for the interaction term and a negative but statistically non-significant main effect for *IT investments*. The results from Table 3 show the benefits from IT investments for banks’ risk assessments during a structural break, such as the COVID-19 pandemic. Without a structural break, we do not find a statistically significant benefit from IT investments on banks’ forecasting accuracy of loan losses. This finding is somehow surprising and contradicts our first expectation. Overall, these results show that IT investments are not significantly beneficial in normal times but allow banks better assess their loan risk in situations of greater uncertainty.

In non-pandemic times, one standard deviation increase in IT investments reduces the dispersion of |*ALLP*| by approximately 3%.^4^ Since the coefficient is not statistically significant, we cannot rule out the possibility that this result occurred by chance. However, one standard deviation increase in IT investments reduces the dispersion of |*ALLP*| by slightly more than 12% in the pandemic period. Consequently, banks with *IT investments* that are one standard deviation higher can estimate 1 out of 10 loan impairments more accurately in economically unstable times.

### Pre-treatment balance between treatment and control group

**Fig. 1:**
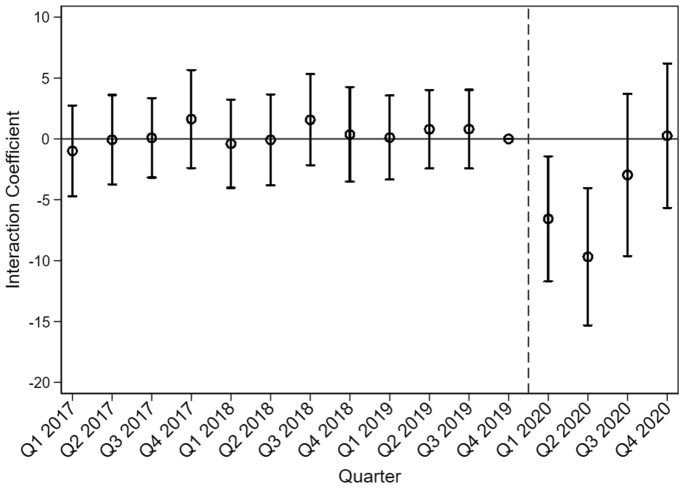
Course of the interaction coefficient of IT Investments and the current quarter over time

Diverging trends in the pre-pandemic period are a major concern for our empirical identification strategy. Consequently, if banks with high and low IT investments show large differences in the trends for |*ALLP|* already long before the crisis, our model would not capture the time trends appropriately. Similarly, if the trend divergence does not happen around the outbreak of the COVID-19 pandemic but long after that, whether it would be responsible for the observed changes in |*ALLP*| would be unclear. Figure 1 shows the interaction coefficient of IT investments and the respective quarter. It clearly shows that the coefficient is not significantly different from zero before the pandemic hit the economy. The first time a negative coefficient is visible, is in the first two quarters of the year 2020. Furthermore, the pre-pandemic standard errors are definitely smaller than in the year of the pandemic. Even though higher standard errors within a pandemic are not surprising, the coefficients before the pandemic has occurred are quite precisely estimated and not statistically significant from zero. Furthermore, we see that the benefits of banks with higher IT investments diminish in the following quarters. This diminishment harkens to the Bayesian learning theory—explaining why we do not find support for IT benefits in stable times. We argue that banks with higher IT investments generally estimate their LLP more precisely and adapt more quickly than banks with lower IT investments. Nevertheless, the latter can adapt to the new situation and learn from the past quarters after the structural break, too, just more slowly than banks with higher IT investments. This newly gained experience then helps banks more precisely estimate the LLP in the upcoming (and eventually more similar) periods, and the difference between banks with higher and lower IT investments diminishes (again) over time.

Figure 1 shows the course of the interaction coefficient of IT Investments and the respective quarters over time from the beginning of the year 2017 to the end of 2020. The circles describe the average coefficients and the corresponding lines represent the standard errors. We use the fourth quarter of 2019, the last quarter in non-pandemic times, as our reference group.

**Fig. 2:**
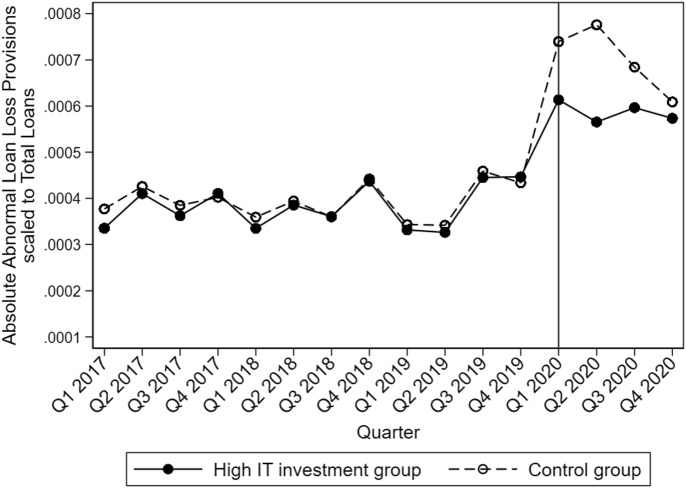
Absolute Abnormal Loan Loss Provisions for banks with respect to their IT investments (median split)

An alternative representation of the parallel trend can be found in Fig. 2. It displays the result of a median split in *IT investments* where we observe both groups’ mean |*ALLP|* and not only the interaction coefficient. Before the outbreak of the COVID-19 virus in the US, |*ALLP|* is very close to each other, independent of the respective IT group. After the COVID-19 outbreak, we see an increase in |*ALLP|* for both groups, which is not surprising due to the increasing uncertainty in economic outlooks and borrowers’ situations. However, this increase in *|ALLP|* is much lower for banks belonging to the high IT investments group. Therefore, banks with more IT investments can better assess their loan risks and better react to changing environments or structural breaks. Figure 2 supports the results of our multivariate regressions that there is indeed a significant difference between banks that invest more in IT and banks that do not. In line with the multivariate regressions, higher IT investments have a decreasing influence on the dispersion of the LLP; in other words, they have a positive correlation with higher quality assessments of loan risk. Again, we see that the benefits of banks with higher IT investments diminish in the following quarters.

Figure 2 shows the course of |ALLP| over time from the beginning of the year 2017 to the end of 2020. We split the sample into two groups. One group contains banks with above-median, and the other group contains banks with below-median IT investments. The solid black line describes the average |ALLP| for banks with above-median IT investments, and the dashed line represents the average |ALLP| for banks with below-median IT investments. The horizontal solid black line marks the first quarter of 2020, which we define as the first quarter in the COVID-19 pandemic in the US.

### Robustness tests

Placebo tests are a possible instrument to test the validity of a Difference-in-Differences approach (Cunningham 2021). Our results could be influenced by other confounding events that happened around the outbreak of the COVID-19 pandemic or are attributable to general time trends. In Fig. 1, we can already see these placebo tests. Since the interaction coefficient between our IT measure and the respective quarters is not significantly different for any period before the outbreak of the pandemic, we conclude that our finding is only valid for the first two quarters of the pandemic timeframe. Overall, the results from the placebo tests show that our main empirical results in Table 3 cannot be attributed to other events but most likely come from the COVID-19 pandemic.

Additionally, our empirical identification strategy is exposed to various concerns, for example, potential endogeneity between the main variables of interest. We tackle the different concerns in different ways. The first concern comes from possible anticipation effects. Suppose firms anticipated the COVID-19 pandemic and thus invested more and earlier in IT investments but simultaneously granted different types of loans with lower *|ALLP|*, then endogeneity could bias our results. To address this concern, column (1) of Table 4 shows an alternative specification if we use IT investments from the year 2017 as our instrument for IT capabilities. We argue that banks’ IT investments in 2017 are unlikely to be affected by any anticipations of the COVID-19 pandemic. Our results are qualitatively unchanged compared to the results of our main specification, and IT investments still have a negative and significant coefficient for the period before the COVID-19 pandemic. Moreover, we conduct the regression also with a rolling IT investments measure over the previous eight quarters to account for changes in the IT strategy in banks over time. Since this measure is not static, we gather results from a “fuzzy Difference-in-Differences” approach which has some important implications for the interpretation of the results as explained by Chaisemartin and D’HaultfŒuille (2018). In case there is no stable treatment over time for the control group, they show that the regression results rest on the assumption that the treatment effects are stable and homogenous over time. Since our treatment variable is, even though variable and a rolling measure over time, rather stable, we are still convinced that the results are correct, at least direction-wise.

**Table 4:** Robustness tests: static IT measure from 2017 and rolling IT measure

Dependent variable	(1)	(2)
|ALLP|	IT Investments 2017	IT investments 8 quarter
IT Investments x COVID Crisis	− 4.19339**	− 4.71836**
	(1.84588)	(1.85487)
COVID Crisis	3.05301***	3.34643***
	(0.46704)	(0.5186)
IT Investments	− 1.43388	− 1.56087
	(1.05939)	(1.06815)
*Controls:*		
dNPL	26.97183*	26.74905*
	(14.07151)	(14.06055)
Lagged dNPL	10.35812	10.74867
	(12.95374)	(12.91138)
RegCap	8.83426***	9.05268***
	(2.70599)	(2.72461)
EBLLP	− 74.39943***	− 74.22252***
	(15.23483)	(15.181)
Asset Growth	5.21572***	5.28317***
	(2.01301)	(2.00568)
Lagged Loans to Assets	− 1.5841	− 1.61358
	(1.36324)	(1.36)
Deposits to Assets	− 4.8261**	− 4.76708**
	(2.16755)	(2.15238)
Lagged Deposits to Assets	0.49417	0.38998
	(1.74744)	(1.73191)
Real Estate Loans	− 0.78641	− 0.85831
	(1.01145)	(1.00577)
Commercial Loans	0.3893	0.33273
	(1.58624)	(1.58207)
Retail Loans	− 0.48658	− 0.69663
	(2.99085)	(2.98153)
State & Time FE	Yes	Yes
Observations	8522	8522
R-squared	0.197	0.198

Standard errors are in parentheses

***p < 0.01, **p < 0.05, *p < 0.1

The table contains the results of the robustness tests. There are two OLS regression specifications with standard errors that are robust and clustered on bank-level. The dependent variable is *|ALLP|*. Column (1) shows the results from the robustness test with the static IT Investments from the year 2017. The regression specification itself is similar to the regressions whose results we present in Table 3. Column (2) contains the results from the regression with the rolling IT measure. Again, the specification we use here is the main specification shown in Table 3. Each column contains data for the entire sample.

Furthermore, functional form misspecification might bias our results. If banks with high IT investments structurally differ from those with low IT investments, and if the control variables cannot adequately capture these differences, Shipman et al. (2017) show that the empirical results might be biased. To alleviate the concerns of functional form misspecification, we conduct a propensity score matching (PSM) analysis. The PSM can be used to estimate causal treatment effects (Caliendo and Kopeinig 2008) and is also a powerful tool if there is misspecification of the functional form in the regression model (Shipman et al. 2017). In our case, the PSM matches banks with comparable, observable variables to generate a control group as similar as possible to the treatment group. The treatment in our framework is whether banks belong to the 50% of banks that spend more than the median bank on IT investments. The first step is to conduct a probit regression of our treatment variable. The result of this regression is provided in Table 5. We include bank-level as well as geographic variables as control variables in our probit regression specification: the amount of *LLP* is scaled to total loans, the *Regulatory Capital* ratio, the amount of *NPL* is scaled to total loans, the *Loans to Assets* and *Deposits to Assets* ratio, the *Size* of the bank, and the *EBLLP*. We also add the *Strictness* Index developed by Agarwal et al. (2014). This index captures the differences between states and their regulatory strictness. Lastly, we add quarter-fixed effects.

**Table 5:** Propensity score matching−first stage and average treatment effect on the treated

Above-median IT Investments	Coef		St.Err	*t* value		*p* value	[95% Conf		Interval]
LLP	1.186		13.08	0.09		0.928	− 24.452		26.823
RegCap	1.268***		0.446	2.84		0.004	0.394		2.142
Strictness	0.591**		0.29	2.04		0.042	0.022		1.161
NPL	− 3.618*		2.032	− 1.78		0.075	− 7.601		0.365
Loans to assets	0.124		0.164	0.76		0.448	− 0.197		0.445
Deposits to assets	1.155***		0.22	5.24		0	0.723		1.587
Size	− 0.181***		0.016	− 11.64		0	− 0.211		− 0.15
EBLLP	1.024		1.14	0.90		0.369	− 1.21		3.258
Loans	0***		0	3.47		0.001	0		0
Fed Chartered	0.141***		0.036	3.94		0	0.071		0.212

Mean dependent var		0.497			SD dependent var			0.500	
Pseudo r-squared		0.027			Number of obs			8398	
Chi-square		311.633			Prob > chi2			0.000	
Akaike crit. (AIC)		11,352.094			Bayesian crit. (BIC)			11,429.487	

ALLP	Treated		Controls		Difference	S.E	T-stat
Unmatched	4.3422		4.664		− 0.3219	0.1022	− 3.15
ATT	4.3387		4.693		− 0.3542	0.1487	− 2.38

		Off Support		On Support			Total
Untreated		1		4226			4227
Treated		29		4142			4171
Total		30		8368			8398

***p < 0.01, **p < 0.05, *p < 0.1

The upper part of Table 5 shows a probit estimation result. The dependent variable is a binary variable indicating whether a bank belongs to the group of banks that invests more than the median bank in IT this is indicated by a value of 1. With this probit estimation, we conduct the Propensity Score Matching. The result of the Propensity Score Matching is shown in the middle part of Table 5. Both groups, namely high IT investment banks (Treated) and low IT investment banks (Control), are then compared. The ATT Difference, together with the respective T-statistic, is the relevant item. ATT stands for average treatment effect on the treated

Our matching procedure allows for the replacement of the control group observations. According to Shipman et al. (2017), this procedure leads to better quality matching. For the matching, we use a caliper width of 0.2, as suggested by Wang et al. (2013). The balancing property between the two groups is satisfied that indicates they significantly differ in the treatment variable but not in the variables for which we carried out the matching.

Again and in line with our previous findings, we calculate a highly significant and negative coefficient for the ATT, stated in Table 5. ATT describes the so-called average treatment effect on the treated−or in our case, the effect of having above-median IT investments. To summarize, a negative ATT is in line with our previous results and strengthens them.

In addition, tax incentives might provide a confounding effect for our sample of banks. Since banks can use LLP to manage earnings and their regulatory capital, we test whether our results hold when we consider tax incentives for banks. For this purpose, we first exclude firms that had losses in a single period from our regression model and observe that our results still hold and remain qualitatively unchanged. The same applies when considering different firm types–namely, whether a bank is a C- or S-corporation. C- and S-corporations differ in their tax regulations because C-corporations are taxed under subchapter C of the IRS and S-corporations are taxed under subchapter S. Thus, we add a triple interaction term to our regression that gives us the coefficient for the firm types during the pandemic that is interacted with *IT Investments*. The unreported results from the triple interactions show that our results are robust for the different tax statuses of banks. While the interaction term between *IT investments* and the pandemic indicator is qualitatively unchanged, the coefficient for the (triple) interaction term is insignificant. Also, the single coefficient for S-corporation banks is, though negative, not significant. Thus, we do not observe a significant coefficient regarding tax motives for our IT investment results and, accordingly, do not expect tax motives to be a driver of banks’ IT investments.

## Limitations

By using standardized data on banks’ IT provided by the FDIC, we gather results supporting the positive value of IT for their loan risk assessments. However, as explained previously, we are only able to retrieve the so-called value capability (Bhatt and Grover 2005). Since IT capability is not only determined by value capability, we cannot make statements about banks’ competitive or dynamic capabilities and their relation to banks’ loan risk assessments. Nevertheless, we expect our measure to provide a conservative estimate of the actual effect of IT capabilities on banks whenever the failure probability does not systematically differ from our dependent variable. Only if the factors that drive the success of IT projects also enable banks to better forecast their loan portfolios in crisis situations could they affect our empirical results. However, it would definitely be insightful to empirically disentangle the different types of IT capabilities and reveal their relation to banks’ loan risk assessments.

## Discussion and conclusion

In this study, we raise the question of the benefits from IT investments for banks. While other studies find diverging results, we provide new empirical evidence and show that IT investments indeed make sense for banks, at least from a loan risk perspective, which is crucial for commercial banks. We investigate whether banks with more IT investments can more precisely estimate their loan risks and thus, assess their loan loss provisions as exactly as possible. While assessments of loan risk in normal times can be anticipated quite well due to low uncertainty in the economic environment, we do expect a benefit from additional IT during normal times, but not a huge one. This is why we include a period of economic distress in our analysis, namely the COVID-19 pandemic. The structural disruption caused by the pandemic creates new dynamics in the economic and business environment that have made it necessary for banks to estimate their loan risks without being able to draw on experience from previous periods.

The final sample of this study comprises 8522 bank-quarter observations from 665 banks. Our study shows that IT investments are indeed helpful for banks’ loan risk assessments in times of economic distress. Banks with higher IT investments estimated loan risks more precisely in terms of LLP than banks with a lower level of IT investments which means that IT investments are unquestionably relevant for banks from a loan risk perspective. Contradicting our first expectation, IT investments are not statistically or significantly beneficial in normal times when speaking in terms of loan risk assessments. This is surprising at first, but keeping in mind that banks can use their experience from previous quarters when no structural break occurs, it appears plausible and relatively straightforward. When no structural disruption occurs, banks can update their expectations and estimations with experience from a similar business environment from past quarters regardless of their IT investments which means they could use last year’s LLP as an anchor and adjust according to the managers’ gut feelings.

The results from this study have important consequences for banks and their digital transformation. First, banks should invest more in IT, even though it might not be profitable immediately. IT investments or capabilities make the bank more resilient to external shocks and structural breaks. Therefore, higher IT investments, or digital transformation in general, are crucial for the stability of the overall banking system. As data becomes more extensive and detailed, there is no other option than to implement or build a business structure that allows more and more data to be used and processed for many bank-specific tasks.

## Data Availability

All data is publically available as indicated in the paper.

## Footnotes

1 Mike Dargan, global head of group technology at UBS: “We had four main focus areas, remote working to enable the employees at UBS, system stability, as we saw a lot of volatility, cyber security, and operations continuity” (Reuters 2021).

2 We regress loan loss provisions on their determinants which we gather from the respective financial statement. *|ALLP|* are the absolute values of the residuals of this regression model. Higher *|ALLP|* indicate a lower quality risk assessment.

3 Banks are only required to report IT investments that exceed $100,000 or 7% of other non-interest expenses. Because we cannot observe the exact value of IT investments for these banks, these observations would dilute the results.

4 We calculate this dispersion by multiplying the standard deviation of *IT Investments* with the sum of the coefficients for the IT main- and interaction-effect and divide the effect by the standard deviation of *|ALLP|*. For the evaluation in the pre-pandemic time, we only consider the main-effect coefficient.
